# Hormones and monensin use to improve pregnancy rates in grazing lactating beef cows in the semiarid region of Argentina

**DOI:** 10.21451/1984-3143-2017-AR0032

**Published:** 2018-08-16

**Authors:** Pablo Sebastián Reineri, Mónica Belén Piccardi, José Ignácio Arroquy J, Arnaldo Fumagalli, María Sumampa Coria, Olegario Hernández, Gabriel Bó, Gustavo Adolfo Palma

**Affiliations:** 1 National Institute of Agricultural Technology, EEA-Santiago del Estero, Santiago del Estero, CP .; 2 Faculty of Agronomy and Agribusiness, National University of Santiago del Estero (UNSE), Santiago del Estero, CP .; 3 Animal Production Laboratory, Institute of Bionanotechnology of the NOA (INBIONATEC), Santiago del Estero, G4206XCP, .; 4 Faculty of Agricultural Sciences, National University of Córdoba (UNC), Córdoba Capital, CP .; 5 Institute of Animal Reproduction Córdoba, Paraje Pozo del Tigre, Córdoba, CP .

**Keywords:** hormone, monensin, pregnancy rate, volatile fatty acids

## Abstract

The objectives of this study were 1) to determine the effect of monensin treatment, alone or combined with a hormonal estrus synchronization treatment, on the pregnancy rate of lactating beef cows, and 2) to evaluate the effect of monensin capsule administration on ruminal metabolism in steers. In experiment 1, ninety-four cows were selected from a 300 cow herd. The experimental design used was a 2 x 2 factorial with the administration of monensin capsule as first factor (M1 = with monensin *vs*. M0 = without monensin) and hormonal treatment as second factor (H1 = with hormonal treatment *vs*. H0 = no hormonal treatment). Thirty-eight days before the beginning of the breeding season, cows were randomly assigned to the first factor, and thirty days later to the second factor, resulting in four treatments: M1H0, M1H1, M0H0 and M0H1. Cow were exposed to bull (bull/cow ratio 1:20) from day 0 (day 0 = start of the breeding season and 38 days after monensin capsule administration) to day 50. Pregnancy diagnosis was performed at 30, 60 and 80 days after start breeding season by ultrasonography. In experiment 2, eight ruminally cannulated crossbred beef steers were randomly assigned to two treatments (M1 and M0). To determine proportion of volatile fatty acids (VFA), ruminal fluid samples were taken on days 0, 40 and 77 of the experimental period, at 0, 4 and 12 h after grazing. In experiment 1, treatments whit monensin did not improve pregnancy rate (P = 0.95), however, hormonal treatment resulted in grater pregnancy rates (P = 0.03). In experiment 2, the proportion of VFA in ruminal fluid of steers was significantly different between treatments. The highest proportion of propionate was found in ruminal fluid from M1 treatment at 12 h after grazing (P = 0.04). In conclusion, the treatment with monensin increased the proportion of propionate. The result might suggest that energy balance was improved in steers, without improvement in cow´s pregnancy rates. Treatment with monensin alone did not improve pregnancy rate, nor did treatment with monensin enhance the pregnancy rate when a hormonal synchronisation treatment was given. Nevertheless, the use of a hormonal treatment increased pregnancy rate, suggesting that it could be used as a suitable tool to enhance the productivity in cows with marginal body condition score.

## Introduction

Anestrus at the beginning of the breeding season is often the main constraint in pregnancy levels of breeding herds. Low forage availability, low body condition score (BCS) at calving and increased requirements for lactation, are the main nutritional causes that directly influence the productivity of the herd. These factors generate a negative energy balance, determining postpartum anestrus ( Diskin *et al*., 2003 ; Hess *et al*., 2005 ; Diskin and Kenny, 2016 ). Also the inadequate glucose availability affects negatively gonadotropin-releasing hormone (GnRH) and the luteinizing hormone (LH) release ( Wettemann *et al*., 2003 ; Hess *et al*., 2005 ), essential hormones for resumption of cyclcicity. The main source of energy in ruminants is obtained from volatile fatty acids (VFA), particularly propionic acid ( Dicostanzo *et al*., 1999 ). Cattle grazing native pasture fed low quality diets, with higher levels of acetate and lower proportion of propionate ( Hawkins *et al*., 2000 ). In these systems, the use of modifiers to manipulate rumen fermentation such as monensin, could stimulate the production of gluconeogenic compounds, changing the proportion of VFA; mainly the proportion of acetate-propionate, towards a higher proportion of propionate, increasing the synthesis of hepatic glucose and improving the energy balance ( Ipharraguerre and Clark, 2003 ). The increase in the proportion of ruminal propionic acid and the availability of glucose, could improve the partition of nutrients towards the hypothalamic-pituitary-gonadal axis, reversing the postpartum anestrus situation ( Diskin *et al*., 2003 ; Hess *et al*., 2005 ). In addition, some authors reported that monensin supplementation decrease time to the first postpartum ovulation in Holstein cows, and increase follicle size at 55 days postpartum in Nellore cows ( Tallam *et al*., 2003 , Matos *et al*., 2004 ). Many studies have been carried out in order to evaluate the use of hormonal treatments to reduce the calving-conception interval ( Bó *et al*., 2003 ; Baruselli *et al*., 2012 ). However, few studies have evaluated the concomitant effect of reproductive hormonal treatments in conjunction with ruminal fermentation modifiers. Therefore, the aims of this study were to evaluate the effect of monensin treatment, alone or combined with a hormonal estrus synchronization treatment, on the pregnancy rate of lactating beef cows and to evaluate the effect of the monensin capsule on ruminal metabolism in steers.

## Materials and Methods

The experiments were carried out over a period of 118 days, on a commercial herd, located in northwest Argentina (S27° 17'34, 3”-W062° 15'14, 1”) during December to March. During the experimental period (from day -38 to 80), the precipitation was 825 millimeter and the average temperature was 26.5°C. The mean maximum and minimum temperature were 32.5°C and 20.5°C, respectively, and the average maximum temperature of the days when the animals were expected to be in estrus after synchronization, (day 0 to 5), was 36.2°C. The animals used in the present study had been grazed in the same paddock on Guinea grass (*Megathyrsus maximus*, cv. Gatton panic) throughout the experimental period. With an initial forage availability of 5763 ± 1359 kg DM/ha (60% stem, 20% green leaf, 2% inflorescence and 18% dead leaf and weeds). Animal handling and experimental procedures were in accordance to institutional protocols for Experimental Animal Care and Use approved by the National Institute of Agricultural Technology (Instituto Nacional de Tecnología Agropecuaria - INTA, 2013 ).

### Experiment 1

**Figure 1:**
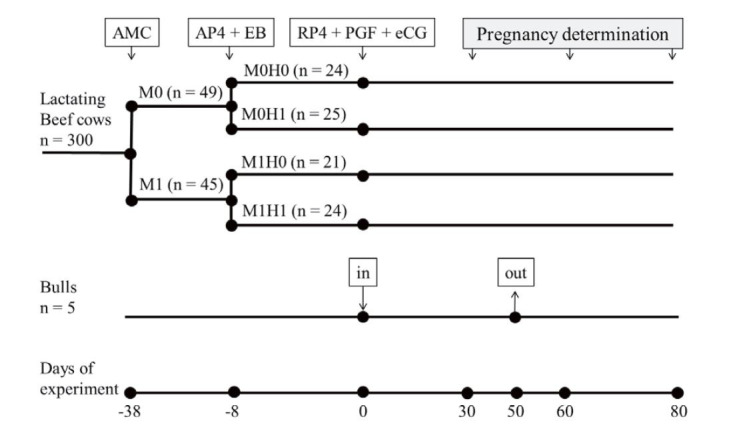
Protocol experiment 1. The experiments were carried out over a period of 118 days. Lactating beef cows were randomly assigned to one of four groups. Ninety-four cows were selected from a 300 cow´s herd. The experimental design used was a 2 x 2 factorial with monensin administration as first factor (M1 = with monensin *vs*. M0 = without monensin) and hormonal treatment as second factor (H1 = with hormonal treatment vs. H0 = no hormonal treatment). Thirty-eight days before the beginning of the breeding season, the cows were randomly assigned to the first factor, and thirty days later to the second factor, resulting in four treatments: M1H0, M1H1, M0H0 and M0H1. Bulls were exposed to cows from day 0 to 50 (bull/cow ratio 1:20) and confirmation of pregnancy was performed at 30, 60 and 80 days after start breeding season by ultrasonography. AMC: administration of monensin capsule; AP4 + EB: intravaginal application of progesterone release device (0,5 g Progesterone, DIB, Syntex, Argentina) for 8 days, plus 2 mg of estradiol benzoate (Gonadiol, Syntex, Argentina) administrated intramuscularly; RP4 + PGF + eCG: progesterone release device was removed (start of natural service), and intramuscularly were injected 0.15 g of D (+) cloprostenol (PGF, Syntex, Argentina) and 400 IU of equine chorionic gonadotropin (Novormon Syntex, Argentina).

Thirty-eight days before the breeding season, crossbreed Zebu (Brangus x Braford) multiparous cows (n = 94) with lactating calves from 3 to 4 weeks of age were selected from a herd of 300 cows ( Fig. 1 ). The selection criteria were based on the absence of corpus luteum, diagnosed by ultrasonography and body condition score (BCS) of 3.84 ± 0.04, (mean ± standard error of the mean; SEM). The BCS was evaluated by optical observation in day -38, 0 and 80, using the score range 1 - 9, 1 = emaciated and 9 = obese ( Richards *et al*., 1989 ).

Cows were randomly assigned to two treatments: M1 treatment (n = 45), with intra-ruminal slow release monensin capsules administered, each capsule containing 32 g of crystalline monensin (Rumensin® Elanco Animal Health, Argentina); and M0 (n = 49) without monensin capsule administered. Thirty days later (day -8), both groups were assign to a second treatment factor (hormone administration) in a 2 x 2 factorial arrangement within a completely randomized design. The H1 group received an intravaginal progesterone releasing device containing 0.5 g of progesterone (DIB, Syntex, Argentina) for 8 days, plus 2 mg of estradiol benzoate (Gonadiol, Syntex, Argentina) administered intramuscularly. On day 0 (breeding season), DIB inserts were removed and 0.15 g of D (+) cloprostenol (PGF, Syntex, Argentina) and 400 IU of equine chorionic gonadotropin (Novormon Syntex, Argentina) were administered intramuscularly. Group H0 did not receive any hormonal treatment.

The ninety-four cows were allocated to each treatment and the factorial combination was as follows: M0H0 (n = 24), M1H0 (n = 21), M0H1 (n = 25) and M1H1 (n = 24).

The bulls used in this study for service were previously evaluated and approved by sanitary, morphological and reproductive standards, in a proportion of bulls to cows of 1:20. Cows were exposed to bulls from day 0 to 50 ( Fig. 1 ). Cows were evaluated for pregnancy and embryonic losses by ultrasonography at 30, 60 and 80 day after start breeding season ( Fig. 1 ), using the ultrasound CHISON 500VET with a 5.0 MHz linear transducer, (Chison Medical Imaging Co., Ltd., Wuxi, China).Positive diagnosis of pregnancy was determined as presence of an embryo or foetus with a visible heartbeat and, at later stages visible foetal movements, total conceptus size, compatible with stage of gestation, and the presence of clear amniotic fluid ( Kastelic *et al*., 1988 ). Embryonic loss or early foetal death was defined as the absence of a viable embryo or foetus on a given day that had been present at the previous examination by ultrasound (Silke *et al*., 2002).

### Experiment 2

**Figure 2:**
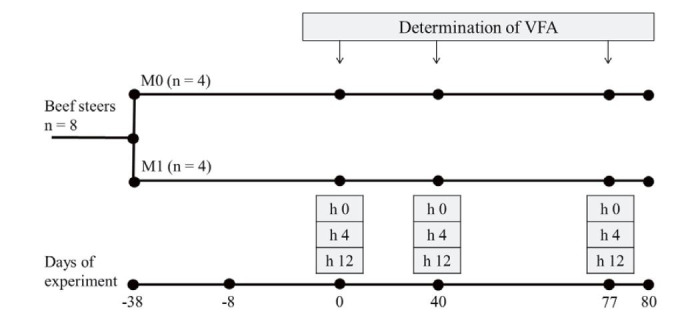
Protocol experiment 2. Eight ruminally cannulated crossbred beef steers, four dosed with monensin (M1) and four without monensin (M0), were used. Ruminal fluid samples were taken through the fistulas on days 0, 40 and 77. Samples were taken at three times during the sampling day, before leaving to graze (hour 0), and 4 and 12 h after the start of grazing. VFA: volatile fatty acids; h: hour.

Thirty-eight days before the breeding season, eight ruminally cannulated crossbred beef steers, four treated with of intra-ruminal slow release monensin capsules (M1**)** and four without monensin capsule (**M0**) were used ( Fig. 2 ). The steers group M1 received a monensin capsule containing 32 g of crystalline monensin (Rumensin® Elanco Animal Health, Argentina). Ruminal fluid samples were taken through the fistulas on days 0 (day 0 = start of the breeding season and 38 days after monensin capsule administered), 40 and 77. Samples were taken at three times during the sampling day, before leaving to graze (hour 0), and 4 and 12 h after the start of grazing ( Fig. 2 ). Twelve hours before each sampling day, the steers were collected from the pasture. At dawn the next day, before the grazing activity, ruminal fluid samples were taken through ruminal fistulas (hour 0), then the steers were allowed to graze for 4 h and then were collected from the pasture to take the second daily sample (hour 4), after sampling they were allowed to graze another four hours to take the last sampling at hour 12 after the start of grazing. For VFA analysis, 8 ml of ruminal liquid were diluted in 2 ml of metaphosphoric acid 25% (w/v), immediately placed at 4°C, and then frozen at - 20°C, to be subsequently analyzed ( Olson, 1991 ). Samples were thawed at room temperature and centrifuged at 17,000 xg for 15 min. The VFA content was analyzed by gas chromatography using a flame ionization detector (Konik HRGC-3000C) fitted with a capillary column Zebron ZB-FFAP (15 mx di 0.32, 0.25 m; Phenomenex). Temperature was set at 100ºC for 3 min, with increase of 8°C/min from 100 to 230°C. The carrier gas was N2 at 1.66 ml/min. Split ratio: 20:1.

### Statistical analysis

Pregnancy rate cow’s data was analyzed as a completely randomized design with a 2 x 2 factorial arrangement and data from BCS in cows and steers ruminal fermentation profile were analyzed as a completely randomized design. To compare the percentage of pregnant animals related to treatments among days of breeding, survival analysis of Kaplan and Meier was used. The difference of the curves given by the treatments was compared using the Log-Rank statistic ( Kaplan and Meier, 1958 ). A high value of Log-Rank corresponds to a small P-value. BCS data from cows of experiment 1 and VFA data from steers of experiment 2 were analyzed as repeated measures with linear mixed-effects model procedure through the R interface with Infostat software ( Di Rienzo *et al*., 2017 ).

The model used for analysis of variance was:

$$Y_{ijkl}=µ+M_{i}+D_{j}+A_{k}+t_{l}+{\left(MD\right)}_{ij}+{\left(Mt\right)}_{il}+{\left(Dt\right)}_{jl}+{\left(MDt\right)}_{ijl}+\varepsilon_{ij}$$

Where *Y*_ijkl_ is the dependent variable (BCS or VFA), µ is the overall mean, M_i_ is a fixed effect of monensin administration i, D_ j_ is a fixed effect of day j_,_ A_k_ is the random effect of animal k, t_l_ is the fixed effect of hour l_,_ (MD)_ij_ is the fixed effect of interaction between monensin administration i and day j, (Mt)_ik_ is the fixed effect of interaction between monensin treatment j with time k, (Dt)_jl_ is the fixed effect of interaction between day j and time l, (MDt)_ijl_ is the fixed effect of interaction among monensin administration i, day j and time l, and ɛ_ ijkl_ is the random error.

In all statistical analyzes used, the level of significance was set at P ≤ 0.05. The trend was considered when the P value was between 0.10 and 0.05.

## Results

### Experiment 1

**Figure 3:**
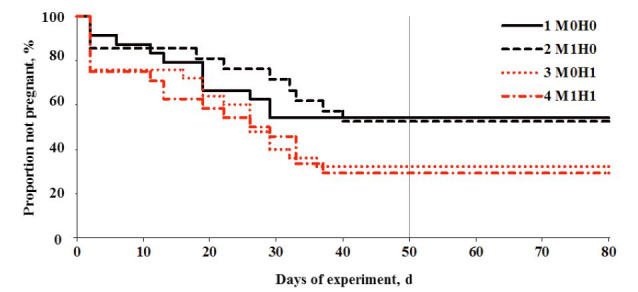
Effect of monensin capsules and hormone treatment on survival curves (n = 94 cows) throughout d 0 to 80 of experiment interval (P = 0.20). ^1^M0H0 = Group treatment control (without monensin and without hormones), ^2^M1H0 = Group treatment with monensin and without hormones, ^3^M0H1 = Group treatment without monensin and with hormones, ^4^M1H1 = Group treatment with monensin and with hormones. Period of exposure bulls with cows from day 0 to 50.

Survival curves for pregnancy rate throughout days of service after hormonal and monensin treatments are shown in Fig.3 . Survival curves, which indicate the percentage of non-pregnant animals as function of time elapsed from day 0 to d 80 not shows significant differences (Log Rank Test = 4.63, P = 0.20). Pregnancy rates on day 80 were: 46% (11/24), 48% (10/21), 68% (17/25) and 71% (17/24) for M0H0, M1H0, M0H1 and M1H1, respectively.

Pregnancy rate was not affected by the interaction between the monensin capsule administration and the hormonal treatment. Therefore, the main effects for monensin and hormonal treatments were evaluated. No difference was observed between M0 and M1 (Log Rank Test = 0.03, P = 0.95; Fig. 3 ). However, statistical difference was observed between H0 and H1. Pregnancy rate of animals without hormonal treatment (H0) was significantly lower than those with hormonal treatment (H1; Log Rank Test = 4.59, P = 0.03, Fig. 3 ) in survival curves. In addition, in none of the treatments evaluated during the experimental period were embryonic losses observed.

Significant differences were found between days of experiment in body condition score, indicating the higher scores at day 80, (day 0 = 4.28 ± 0.04 and day 80 = 5.57 ± 0.12; mean ± SEM; P < 0.0001). Nevertheless, cow BCS not shows statistical differences at the end of breeding season among treatments (M0H0 = 5.63 ± 0.10, M1H0 = 5.36 ± 0.15, M0H1 = 5.56 ± 0.10 and M1H1 = 5.76 ± 0.14; P = 0.37), or among the main effects (M0 = 5.6 ± 0.07, M1= 5.57 ± 0.10, P = 0.14; H0 = 5.53 ± 0.08, H1= 5.65 ± 0.08, P = 0.76).

### Experiment 2

**Figure 4:**
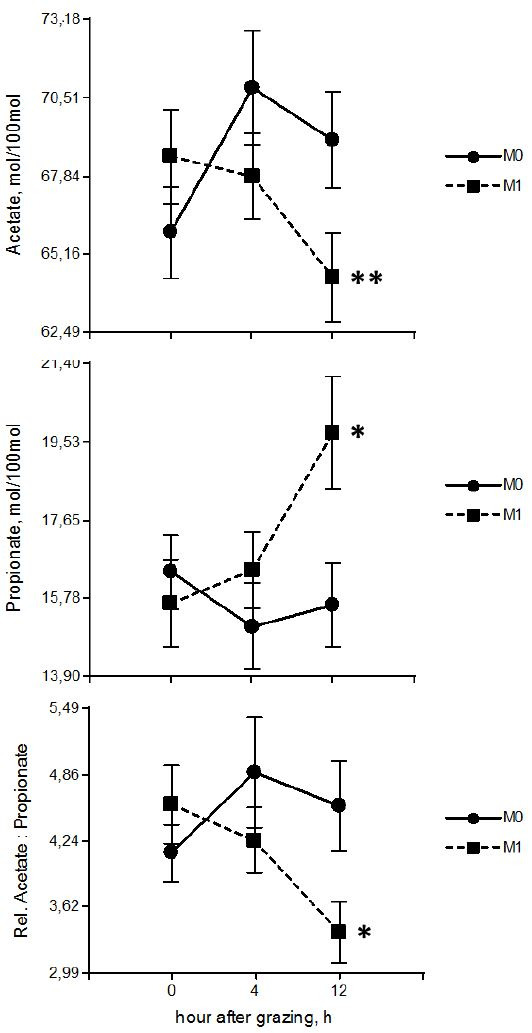
Effect (mean ± SEM) of treatment x hour after grazing interaction on the VFA proportion. The proportion of acetate and propionate and the acetate: propionate ratio values presented expresses the average of the three sampling days (0, 40 and 77). Samples were taken at three times during the sampling day (0, 4 and 12 h after grazing). M1 = with monensin and M0 = without monensin. *P < 0.05; **P < 0.10 and >0.05).

The molar proportion of volatile fatty acids with statistical significant differences was detailed in Fig. 4 , data expresses the average for the three sampling days (0, 40 and 77). Acetate proportion was not affected by any of the variables studied (treatment [P = 0.37], day [P = 0.82], hour [P = 0.26], interactions between treatment x day [P = 0.71], treatment x hour [P = 0.06] and among treatment x day x hour [P = 0.43]), there was a tendency in the interaction between treatment x hour, resulting in lower level of acetate in M1 group at 12 h after grazing (63.55 ± 2.02 *vs*. 68.96 ± 2.11 mol per 100 mol [mean ± SEM], P = 0.06). No statistical differences were observed in proponiante proportion between treatments (P = 0.18), days (P = 0.46), hours (P = 0.06), interactions treatment x day (P = 0.75), treatment x day x hour (P = 0.15). However, statistical difference was observed in the interaction between treatment x hour, propionate proportion was modified by the ruminant modulator in steers at 12 h after grazing, the group with monensin administration (M1) produced higher levels of propionate than M0 (20.05 ± 1.35 *vs*. 15.25 ± 1.41 mol per 100 mol [mean ± SEM], P = 0.02, respectively). The acetate: propionate ratio did not show significant differences between treatment (P = 0.20), day (P = 0.94), hour (P = 0.20), interaction between treatment x day (P = 0.89) and among treatment x day x hour (P = 0.319). However, statistical difference was observed in the interaction between treatment x hour, the acetate: propionate ratio was higher in animals without monensin administration than animals with the ruminant modulator (4.73 ± 0.49 *vs*. 3.23 ± 0.47 mol per 100 mol [mean ± SEM], P = 0.04, respectively).

## Discussion

Nutritional management plays a very important role in reproductive programs. Body energy reserve at calving is the most important factor influencing pregnancy rate in beef females. Energy and protein are the nutrients required in the greatest amounts and are the first priority in nutritional programs to optimize reproduction ( Bossis *et al*., 1999 ; Diskin *et al*., 2003 ; Hess *et al*., 2005 ; Diskin and Kenny, 2016 ). Beef females underfed or in poor body condition lack ovarian activity as a result of suppression of pulsatile release of LH under the control of GnRH ( Rasby and Funston, 2016 ). Feeding monensin to grazing beef cows did not affect BCS change in experiment 1, however differences between days were observed, and founding higher scores on day 80 of the experiment. In previous literature, cows consuming monensin during gestation had decreased forage intake, accompanied by either an improvement ( Sexten, 2011 ) or no change in cow gain ( Linneen *et al*., 2015 ). Bretschneider *et al*. (2008) reported an increase in average daily gain of 12.1% for growing cattle consuming monensin when comparing 46 experiments in a review on the effects of feeding grazing cattle monensin. Perhaps, in this study, monensin simply did not improve energetic efficiency in large enough magnitude to elicit a gain response.

It has been demonstrated that limited metabolic fuel availability, promotes an inadequate production and release of hypothalamic GnRH and pituitary LH ( Wettemann *et al*., 2003 ; Hess *et al*., 2005 ). In this sense, low levels of LH, results in low production of androgens and consequently low concentration of estrogen. In this scenario, not pre-ovulatory LH peak is produced, and dominant follicle could suffer atresia, resulting in a new anovulatory follicular wave ( Roche *et al*., 1992 ). This anestrous situation, in low BCS cows, could be reversed by administration of exogenous hormones, such as estradiol, GnRH, LH and eCG ( Bó *et al*., 2003 ; Sá Filho *et al*., 2010b , Baruselli *et al*., 2012 ). Furthermore, it has been suggested that exogenous hormonal stimulation is necessary to achieve adequate follicular growth, successful ovulation of competent oocyte, and the subsequent formation of functional corpus luteum able of maintain pregnancy ( Bó *et al*., 2003 ; Sá Filho *et al*., 2010a ; Baruselli *et al*., 2012 ; Núñez-Olivera *et al*., 2018 ). Moreover, previous studies showed that an increase in circulating progesterone concentrations during the first week after ovulation effectively stimulates embryo enlargement and interferon-τ secretion, favoring maintenance of pregnancy. Consequently, high-serum progesterone concentrations during the first 2 weeks of gestation are associated with greater pregnancy rates. Similarly, in the present study, the hormonal treatment could achieve follicular atresia and induce new follicular wave’s development at 3-4 days. Also, removing progesterone device and injecting the prostaglandin F2α (PGF2α) plasma progesterone concentration decreases, increasing the LH pulses frequency.

In previous studies, eCG treatment in lactating beef cows in anestrous or with low body condition score, improved follicular growth, produced larger diameter of dominant follicle, increase ovulation ( Sá Filho *et al*., 2010b ), or increase progesterone concentrations in the next cycle ( Baruselli *et al*., 2004 , 2012 ). Moreover Núñez-Olivera *et al*. (2018) demonstrated that eCG on day 14 after artificial insemination at fixed time (FTAI) produces positive effect on serum progesterone concentrations during maternal recognition of gestation in anestrous beef cows. In addition, the eCG treatment in combination with progesterone device elimination at day 14 significantly improved the pregnancy rate compared to the control group without treatment with eCG ( Núñez-Olivera *et al*., 2018 ). Positive effects eCG on reproduction described above can be supported due to the it has the peculiar property of provoking both follicle-stimulating hormone (FSH) and LH activity on follicle ( Murphy and Martinuk, 1991 ) and corpus luteum ( Stewart and Allen, 1981 ) in non-equid species ( Murphy, 2012 ). The biological basis for this dual activity is believed to be the result of promiscuity of the mammalian FSH receptors, imparting the capacity to respond to this equine LH-like hormone ( Murphy, 2012 ).

In the present study, the use of monensin had no effect on the pregnancy rate in beef cow. Similar results were described by other authors ( Lean *et al*., 1994 ; Hayes *et al*., 1996 ; Beckett *et al*, 1998 ). However, Tallam *et al*. (2003) reported that the first postpartum ovulation occurred earlier in cows fed monensin than in the control group in multiparous Holstein cows and Matos *et al*. (2004) found that monensin increased follicle size at day 54 postpartum, but did not affect ovulation rate of Nellore cows. These differences could be associated with the breed of animals and the type of feeding used in each study.

In experiment 2 monensin capsule increases ruminal propionate proportion (gluconeogenic VFA) in cannulated beef steers grazing, as was suggested by other authors (Ipharraguere and Clark, 2003; Rasby and Funston, 2016 ). The alteration of acetate to propionate ratio was enhanced by the increase in propionic acid production and decrease of acetic acid production, probably caused by the monensin’s ability to modify the ruminal microbial population, benefiting propionic acid producing bacteria ( Russell and Strobel, 1989 ; Bell *et al.,* 2017 ). The increase in propionate concentrations could be followed by an increase in blood glucose levels, and improved GnRH secretion ( Randel, 1990 ; Duffield *et al*.,1999 ). Although ruminal metabolism differences were observed between steers that received monensin supplement and those that did not, it was possibly that propionate levels obtained in cows treated was not enough to affect the pregnancy rate, since the nutritional demands between both categories of animals are different ( Fox *et al*., 1992 ). There are consistent meta-analysis data evaluating the impact of monensin on lactating dairy cow health and reproduction showing no reproductive benefits of monensin on this animal category ( Duffield *et al*., 2008 ), nevertheless there is lack of information or studies concerning the effect of fermentation modulators on beef cow reproduction. This study demonstrates for first time, that the interaction effect of a ruminal fermentation modulator (monensin) and hormone not produces differences in pregnancy rates, whereas hormone administration alone could increase this rate in beef cows with marginal body condition scores during the reproductive season.

In conclusion, the interaction between hormonal and monensin treatment and monensin alone did not generate significant differences in the pregnancy rate. However, in the present study, the main effect of hormonal treatment with progesterone devices, estradiol and eCG increased pregnancy rate on low BCS cows at the beginning of the breeding season. Monensin treatment increased levels of ruminal propionate which could improve energetic efficiency. The use of hormonal treatments would be a suitable tool to increase the pregnancy rate on lactating cows with poor BCS. Although further experiments are essential in order to corroborate these results. It would also be interesting to include some rumen fermentation modifier (monensin or other ones) to enhance some nutritional factors which may interfere and/or limit the use of hormonal treatments and get better reproductive indices.

Conflicts of interest statement
